# Advancing translational research in digital cardiac rehabilitation: The preparation phase of the Multiphase Optimization Strategy

**DOI:** 10.1093/tbm/ibae068

**Published:** 2024-12-17

**Authors:** Eanna Kenny, John W McEvoy, Jenny McSharry, Rod S Taylor, Molly Byrne

**Affiliations:** Health Behaviour Change Research Group, School of Psychology, University of Galway, Galway, H91 EV56, Ireland; School of Medicine, University of Galway, Galway, H91 V4AY, Ireland; National Institute for Prevention and Cardiovascular Health, Galway, H91 FF68, Ireland; Health Behaviour Change Research Group, School of Psychology, University of Galway, Galway, H91 EV56, Ireland; MRC/CSO Social and Public Health Sciences Unit and Robertson Centre for Biostatistics, Institute of Health and Well Being, University of Glasgow, Glasgow, G12 8TB, UK; Health Behaviour Change Research Group, School of Psychology, University of Galway, Galway, H91 EV56, Ireland

**Keywords:** cardiovascular disease, cardiac rehabilitation, digital, Multiphase Optimization Strategy, behavioral science, behavior change

## Abstract

While digital cardiac rehabilitation (CR) is an effective alternative to center-based CR, its components and mechanisms of change remain poorly understood. The Multiphase Optimization Strategy (MOST) provides a framework that allows the effects of individual components of complex interventions to be studied. There is limited guidance within MOST on how to develop a conceptual model. This article describes the development of a conceptual model of digital CR. The conceptual model was developed based on several strands of evidence: (i) a systematic review of 25 randomized controlled trials to identify the behavior change techniques in digital CR interventions, (ii) a qualitative study of patients’ (*n* = 11) perceptions of the mechanisms of digital CR, and (iii) a review of international guidelines. Tools and frameworks from behavioral science, including the Behaviour Change Wheel, Capability, Opportunity, Motivation and Behavior model, and Theoretical Domains Framework were used to integrate the findings. An initial conceptual model of digital CR was developed and then refined through discussion. The conceptual model outlines the causal process through which digital CR can enhance outcomes for patients with cardiovascular disease. The model illustrates the key intervention components (e.g. goal setting and self-monitoring, education, exercise training), targeted outcomes (e.g. physical activity, healthy eating, medication adherence), and theorized mediating variables (e.g. knowledge, beliefs about capability). The article provides an example of how behavioral science frameworks and tools can inform the preparation phase of MOST. The developed conceptual model of digital CR will inform guide decision-making in a future optimization trial.

## Teaser

A case study of developing a conceptual model of digital cardiac rehabilitation as part of the preparation phase of the Multiphase Optimization Strategy (MOST).

**Implications**

**Practice:** The conceptual model guided by the Multiphase Optimization Strategy (MOST) identifies the key components and behavior change techniques for inclusion in digital cardiac rehabilitation.

**Policy:** Poor participation, adherence, and completion of cardiac rehabilitation underscore the need for alternative models of delivering rehabilitation.

**Research:** MOST is a translational research framework that can guide all phases of digital cardiac rehabilitation research, from intervention development through optimization and implementation.

## Introduction

Cardiovascular diseases (CVD) are the leading cause of death worldwide and a major contributor to disability [1]. Their impact on healthcare systems is also significant, with annual direct and indirect costs exceeding $400 billion in the USA alone [2]. It is estimated that 80% of premature heart attacks and strokes are preventable [3], meaning that greater emphasis on CVD prevention and management could save thousands of lives each year.

Cardiac rehabilitation (CR) is a complex, multicomponent intervention that includes exercise training, nutritional counseling, risk factor management, and psychosocial support [4]. Cochrane systematic reviews provide robust evidence that CR can lead to a reduction in CVD outcomes, all-cause hospitalization and improvements in functional status, levels of depression and anxiety, and health-related quality of life [5, 6]. Informed by this evidence, current clinical guidelines from the European Society of Cardiology (ESC) and the American Heart Association and the American College of Cardiology (AHA/ACC) recommend CR referral for patients following acute coronary events, coronary revascularization procedures, or heart failure [7, 8].

Despite the compelling evidence and guideline recommendations, participation, adherence, and completion of CR remain stubbornly low. Data from the UK [9] and Europe [10] reveal that less than half of eligible coronary patients participate in CR programs after an acute event. The USA presents a similar scenario, with a study involving Medicare beneficiaries aged 65 and over indicating that only 29% of patients initiated CR, and of those only 8% completed the recommended full dose of 36 sessions [11]. Barriers to participation can exist at the patient level (e.g. time constraints, work/care commitments, geographical distance), physician level (e.g. lack of referrals), and service level (e.g. resource limitations) [12, 13]. Furthermore, the capacity of center-based CR facilities to offer services to all eligible patients is severely limited. It is estimated that in Europe there is only one program slot available for every seven patients [14], while in the USA, it has been suggested that even if all center-based CR programs operated at 110% capacity, only 45% of eligible patients could be accommodated [15]. Given these challenges, there have been calls for alternative CR delivery models to enhance accessibility, promote engagement, and expand service capacity [16].

One such alternative is the delivery of CR through digital means, using information communication technologies and videoconferencing. Early evidence from systematic reviews suggests that digital CR may be as effective as traditional, in-person rehabilitation at improving CVD risk factors [17, 18] and may promote greater adherence [19, 20]. However, digital CR is an emerging delivery model, and its adoption was rapidly accelerated in response to the suspension of center-based CR during the coronavirus disease 2019 (COVID-19) pandemic [21]. Consequently, there is uncertainty about what makes these programs successful and how they should be structured. Defining the intervention content and identifying active components in CR has been previously identified as a challenge [22]. This is due to the predominant use of single-arm or parallel-group randomized designs in existing CR trials, which, while suitable for evaluating the relative efficacy of one intervention versus another or a control, provide limited insight into the specific mechanisms by which complex interventions operate. Understanding the active ingredients and mechanisms of change of digital CR is necessary to improve the effectiveness of these interventions.

The Multiphase Optimization Strategy (MOST) is an engineering-inspired framework for the development, optimization and evaluation of behavioral, biobehavioral, and biomedical interventions [23]. In contrast to traditional randomized controlled trial (RCT) designs, which test the efficacy of one intervention package against another, MOST involves the use of efficient experimental designs aimed at identifying the “active” intervention components that produce the most significant impact, without exceeding key constraints (e.g. cost, time). MOST comprises three phases: preparation, optimization, and evaluation. In the preparation phase, the focus is on developing a conceptual model, identifying intervention components, conducting pilot and feasibility work, and deciding on the optimization objective—how effectiveness will be balanced against affordability, scalability, and efficiency [24]. The optimization phase involves conducting an optimization trial to assess the performance of candidate intervention components that could be included in the optimized intervention package. Finally, the evaluation phase involves assessing the optimized intervention in a traditional two-armed RCT. MOST is a translational research framework that provides a systematic roadmap for progressing from basic science to translational science [25]. It offers a structured approach to refining and translating behavioral interventions, making it a valuable tool for advancing research and practice across various domains of behavioral medicine. It outlines specific objectives for each phase of the research, facilitating the identification of the next best steps in the process and thereby helping to narrow the gap between research and practice.

This article centers on the preparation phase of MOST, particularly the development of a conceptual model of digital CR. The role of the conceptual model is to express “all of what is known or hypothesized about how the intervention under development is to intervene on the behavioral, biobehavioral, or biomedical process” [23]. In other words, the conceptual model serves as the blueprint for the intervention, specifying the candidate intervention components, the proximal mediators targeted by each component, and the causal pathways through which these components are expected to influence proximal and distal outcomes. The conceptual model represents an important first step in MOST and shapes the choice of experimental design to use in the optimization phase. There is growing interest in conducting MOST studies [26] and also in early-stage research, such as that performed in the preparation phase, as investing time in this stage is vital for ensuring methodological rigor and reducing research waste [27]. Despite this interest, there is limited guidance within MOST on how to develop a conceptual model, and few published papers describing how the preparation phase supports optimization [26].

The aim of this article is to describe the process of developing a conceptual model of digital CR through three interconnected studies: a systematic review of RCTs to identify the effective components in digital CR interventions, a qualitative study of patients’ perceptions of the mechanisms of change associated with digital CR, and a review of international guidelines. This selection of studies was influenced by published guidance on developing complex interventions [28], which recommends reviewing published evidence and undertaking primary data collection. This includes qualitative research to understand what matters most to the target population and the context in which the intervention will operate. In addition to describing the development process, this article aims to illustrate how the Behaviour Change Wheel (BCW) [29, 30] approach can be integrated with MOST during the preparation phase. The BCW framework offers standardized guidance on developing and adapting effective behavioral interventions, with the Capability, Opportunity, Motivation and Behavior (COM-B) model [30] serving at its core. The COM-B model proposes that for behavior change to occur, individuals need the necessary physical and psychological “capability” to perform the behavior, the social and physical “opportunity” to engage in it, and the automatic and reflective “motivation” to do so. Extending the COM-B, the Theoretical Domains Framework (TDF) [31] synthesizes 33 behavioral theories and 128 constructs into 14 distinct theoretical domains of behavior, facilitating the identification of potential sources of behavior change. Complementing the BCW, the behavior change technique taxonomy v1 [32] identifies specific techniques for behavior change. Marques and Guastaferro [33] highlighted that MOST can be integrated with intervention development frameworks, such as the BCW, to improve the effectiveness of digital interventions. They specifically note that the BCW approach can support the development of a conceptual model and the selection of intervention components. By incorporating these tools and frameworks from behavioral science, this article serves as a valuable case study, providing practical insights that can guide future researchers during the preparation phase of MOST.

## Methods

The conceptual model was developed through a comprehensive synthesis of multiple sources of evidence, including a systematic review and meta-analysis, a qualitative study, and a review of international guidelines for CR.

### Systematic review and meta-analysis

We conducted a systematic review of digital CR interventions that aimed to (i) determine the effectiveness of digital CR interventions; (ii) identify the behavior change techniques (BCTs) used in these interventions; and (iii) examine the BCTs and intervention characteristics that were associated with effective digital CR programs. The systematic review has been previously published, with the methodology briefly described here and described in detail elsewhere [17]. The databases PubMed, MEDLINE (Ovid), EMBASE (Elsevier), CINAHL (EBSCOhost), PsycINFO (Ovid), and Cochrane Central Register of Controlled Trials (Wiley) were searched for RCTs of digital CR interventions with patients with any form of heart disease. Data were extracted using the Template for Intervention Description and Replication (TIDieR) checklist [34] and interventions were coded using the BCT taxonomy (v1) [32]. The primary outcomes of interest were changes in health-related behaviors (e.g. physical activity, healthy eating, smoking cessation, and medication adherence). Secondary outcomes included clinical and physiological outcomes. BCTs and TIDieR findings were synthesized narratively, and outcome data were quantitatively synthesized in a series of meta-analyses by outcome using Review Manager (RevMan) version 5.425. BCTs were considered “effective” when there was a statistically significant difference between intervention and comparator in a behavioral outcome.

### Qualitative study

Semi-structured interviews were conducted from March to August 2022 with patients who had participated in one of two digital CR programs offered on the island of Ireland (for more detail on the sample and recruitment see Kenny *et al*. [35]). The aims of the interviews were 2-fold: (i) to explore the experiences of patients who participated in digital CR programs; and (ii) to explore their perceptions of the mechanisms of change associated with digital CR. The findings relating to the first aim have been previously published [35], while those related to the second aim were used to inform the mediators included in the conceptual model and are presented here for the first time. A convenience sampling approach was used in this study. A total of 99 potential participants were invited to participate via an email sent by study partners at the two CR centers. Interested participants then contacted the research team to organize an interview. The interview topic guide is included as a Supplementary File. Interviews were audio recorded, transcribed verbatim, and analyzed using the Framework Method [36]. Both deductive and inductive coding were employed using NVivo 12. Interviews were first coded deductively using the 14-domain TDF [31] to identify the key determinants that influenced behavior change. Where interview text related to more than one domain, it was coded in both, otherwise, it was coded under the domain that most effectively captured the content. Next, extracts coded under each domain were analyzed using inductive content analysis [37] to generate explanatory themes linked to the domain as outlined in Atkins *et al*. [38].

### Review of international guidelines

International guidelines, recommendations, and priorities for the delivery of CR from the AHA/ACC, the ESC, and the British Association for Cardiovascular Prevention and Rehabilitation (BACPR) were selected for review and analysis. These organizations were specifically chosen due to their prominence in offering leading guidelines for CR and CVD prevention. The review identified the core components of CR, providing valuable insights into the current recommendations and standards for the content and structure of each component. This process helped to ensure the development of a comprehensive conceptual model aligned with best practices.

### Synthesis

The findings from the three sources of evidence were synthesized using the TDF [31] and the COM-B [30]. The systematic review identified the BCTs that were frequently used in digital CR and associated with significant improvements in behavioral outcomes. This led to the identification of a candidate list of BCTs capable of influencing target behaviors of interest (e.g. physical activity, healthy eating, medication adherence, smoking cessation). To ensure alignment with the core components of CR, the candidate BCTs were cross-referenced with international guidelines. Next, the qualitative study explored the potential mechanisms of change within digital CR and identified the theoretical domains that could explain how these programs effectively enhanced behavioral outcomes. Links between the identified BCTs and theoretical domains were explored, revealing the relationship between specific techniques and the underlying mechanisms through which they influence behavior. This analysis resulted in an initial conceptual model that visually represented the interplay between BCTs and theoretical domains within the context of digital CR. Feedback on the model was sought from key stakeholders with expertise in CR, intervention development, and behavior change theory. This input was used to refine the model, enhancing its reliability and applicability.

## Results

### Systematic review and meta-analysis

In total, 25 RCTs were included in the review. Table 1 presents a summary of the key findings.

**Table 1:** Summary of the key findings from the systematic review of digital CR interventions [17]

Systematic review findings
Digital CR was comparable to center-based CR on all outcomes.
Compared with usual care, digital CR led to improvements in daily steps, light physical activity, medication adherence, functional capacity, and low-density lipoprotein-cholesterol.
Five interventions delivered all the core components of home-based CR [39] (patient assessment, exercise training, diet management, psychosocial support, medication adherence, and risk factor management).
Features of the interventions included: - Health and lifestyle information (*n* = 21, 84%) - Monitoring of health behaviors (*n* = 23, 92%) - Goal setting (*n* = 18, 72%) - Personalized feedback (*n* = 17, 68%) - Reminders and prompts (*n* = 12, 48%) - Questions and answers with program staff (*n* = 11, 44%)
The quality of intervention description and reporting was poor. Using the items from the TIDieR checklist, we found that the completeness of reporting in the studies ranged from 42% (*n* = 5) to 92% (*n* = 11). The intervention materials (Item 3) were only adequately described in six studies (24%), and the assessment of intervention adherence or fidelity (Item 11) was reported in nine studies (36%).
The risk of bias was low in eight studies (32%), of some concern in 14 studies (52%), and high in three studies (16%).

### BCTs identified in the systematic review

A total of 37 unique BCTs were coded in the interventions. A summary of the most frequently coded BCTs in the interventions is presented in Table 2. In short, they included 2.3 self-monitoring of behavior (*n* = 21; 84%), 2.2 feedback on behavior (*n* = 17; 68%), 5.1 information about health consequences (*n* = 16; 64%), 7.1 prompts/cues (*n* = 14; 56%), and 1.1 goal setting (behavior) (*n* = 13; 52%). When explored by outcome, interventions that effectively improved physical activity frequently included the BCTs 2.3 self-monitoring of behavior, 2.2 feedback on behavior, 5.1 information about health consequences, 1.1 goal setting (behavior), and 3.1 social support (unspecified). Interventions that were associated with improvements in diet frequently used the BCTs 1.3 goal setting (outcome) (*n* = 2, 66%), 2.2 feedback on behavior (*n* = 2, 66%), and 5.1 information about health consequences (*n* = 2, 66%). In relation to smoking cessation, interventions that demonstrated positive results included the BCTs 2.3 self-monitoring of behavior, 5.1 information about health consequences, and 7.1 prompts/cues. Finally, interventions associated with improvements in medication adherence included the BCTs 5.1 information about health consequences, and 7.1 prompts/cues.

**Table 2:** Most frequently included BCTs in the interventions

BCT no.	BCT label	Total *N* = 25 (100%)
2.3	Self-monitoring of behavior	21 (84%)
2.2	Feedback on behavior	17 (68%)
5.1	Information about health consequences	16 (64%)
7.1	Prompts/cues	14 (56%)
1.1	Goal setting (behavior)	13 (52%)
2.4	Self-monitoring of outcome(s) of behavior	10 (40%)
1.3	Goal setting (outcome)	9 (36%)
8.7	Graded tasks	9 (36%)
3.1	Social support (unspecified)	8 (32%)
1.4	Action planning	7 (28%)
1.5	Review behavior goal(s)	7 (28%)
4.1	Instruction on how to perform the behavior	7 (28%)
1.2	Problem-solving	6 (24%)
2.7	Feedback on outcome(s) of behavior	6 (24%)
2.6	Biofeedback	5 (20%)
3.2	Social support (practical)	5 (20%)
9.1	Credible source	5 (20%)
3.3	Social support (emotional)	4 (16%)
6.1	Demonstration of the behavior	4 (16%)
10.4.	Social reward	4 (16%)

### Qualitative study

A total of 11 participants took part in a semi-structured interview. Ten interviews were conducted online via Zoom, and one interview was conducted in person. The mean age of the participants was 64 (SD = 8.27; range 50–75). The majority of participants were male (*n* = 9, 82%), married (*n* = 10, 91%), and had undergone coronary angioplasty.

**Figure 1:**
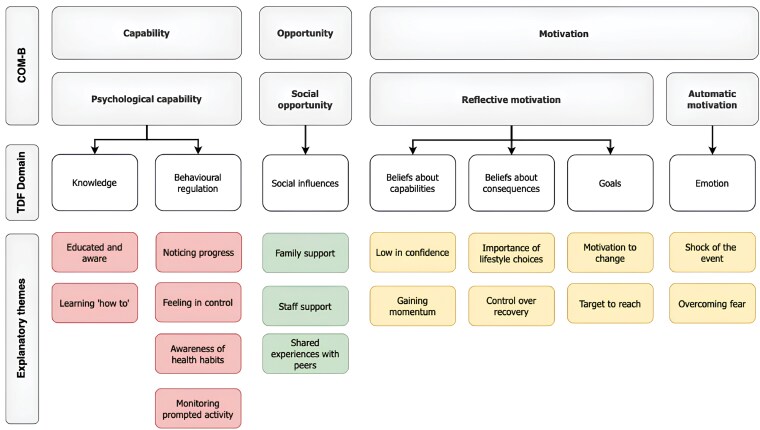
COM-B factors, TDF domains, and explanatory themes identified during the qualitative analysis

Fig. 1 presents a diagram of the COM-B factors, TDF domains, and explanatory themes identified during the analysis. A total of seven theoretical domains were coded in the analysis. The domains related to psychological capability (*knowledge*, *behavioral regulation*), social opportunity (*social influences*), reflective motivation (*beliefs about capability*, *beliefs about consequences*, *goals*), and automatic motivation (*emotion*).

### Domains related to capability

Two TDF domains related to psychological capability were coded from the interviews. Participants frequently highlighted how digital CR programs significantly improved their *knowledge*. The programs enhanced their understanding of CVD, its risk factors, and the impact of their behavior on managing their condition. Additionally, participants acquired practical knowledge on topics such as structuring exercise and distinguishing between healthy and unhealthy food choices. This newfound knowledge empowered them to make positive lifestyle changes, “*I was learning about how to protect my body, how to build up my heart muscle, again, how to do all that kind of stuff, you know*” (P04, Male, 75 years).

Furthermore, participants emphasized the value of *behavioral regulation* in achieving health targets. Actively monitoring physical activity and diet made participants more aware of their health-related habits and allowed them to track their progress toward predefined goals. Monitoring served as a prompt to adjust their behavior if they fell short of their targets, “*if I’m on an office day and I see that I’ve only done 5000 steps, we’ll go for a walk that night or a walk in the morning or a run in the morning before sitting in the office, you know so I will always try and get it*” (P10, Male, 50 years). This practice allowed participants to document their progress and gave them a sense of control over their recovery.

### Domains related to opportunity

The single TDF domain related to psychological opportunity was *social influences*, which featured prominently in the interviews. Participants emphasized that social support played a crucial role in their behavioral change and came from various sources. First, program staff played a crucial role by being available to answer questions, provide reassurance, and put participants’ minds at ease. Additionally, partners and family members were cited as influential in helping participants adapt to a new lifestyle and make behavioral changes, “*it’s never the person in isolation, but the family that has to be involved. And in our case we did. We worked together on improving the diet or making more homemade food*” (P01, Male, 59 years). Lastly, participants emphasized the importance of completing rehabilitation with a peer group as this helped normalize the experience of having a cardiac event and offered an opportunity to share experiences with others, “*But what was wonderful was meeting other people who had a similar experience. Because you feel completely kind of isolated in your experience and other people don’t know what you’ve been through. And there’s a huge emotional component post heart surgery*” (P03, Male, 54 years).

### Domains related to motivation

The TDF domain *emotions* was the only domain related to automatic motivation that featured in the interviews. Participants often described the cardiac event as a shock that left them feeling depressed and fearful. The support provided during the CR program played a crucial role in helping participants address these negative emotions and overcome their fears “*Before that I was worried. I was nervous. And I was suffering from depression as well because I thought I was going to die…it was terrible but they (programme staff) were able to talk me through that as well*” (P04, Male, 75 years).

Regarding reflective motivation, several TDF domains were coded. Initially, participants *believed themselves incapable* of adapting to life after the cardiac event and making the required lifestyle changes. Their lack of confidence was particularly evident when aiming to meet the physical activity requirements of the program, “*I started off with, really wasn’t able to walk very far wasn’t able to, you know, was hit pretty hard by the heart attack like and being able to build that up slowly and having the confidence to do it*” (P08, Male, 57 years). Clear health-related *goals* and specific individualized plans on how to achieve them were essential. Setting goals provided participants with a target to reach and doing so with a member of the program staff added to their motivation, “*once you sit down with somebody and say what you’re going to do you feel very guilty then if you didn’t do it. You know, so it motivated you to keep it up*” (P05, Female, 72 years). As participants reached their targets and progressed through the program, their confidence increased, “*it gains momentum when we behave a certain way over a 2-month period*” (P01, Male 59 years).

Finally, the education and advice provided during the program altered participants’ *beliefs about the consequences* of their behavior. Participants learned how stress, poor diet, and lack of exercise could affect their cardiovascular health, “*Like God I was gone to the rack and ruin before, I didn’t really realize that I was doing so much damage to my body by improper diet, stress. I ran my own company…and that’s really what caused the heart attack was the stress of running that company*” (P04, Male, 75 years). However, they realized that they had the power to change their lives by following the advice from the program staff, “*they put it into your head that this is the best way to do it. And if you do it, it’ll help you a lot and it did*” (P11, Male, 62 years).

### Review of international guidelines

Table 3 presents the international guidelines that were reviewed and describes how each document influenced the development of the conceptual model.

**Table 3:** International guidelines on CR and CVD prevention that informed the conceptual model

Organization	Guideline title	Year	Influence on conceptual model
American Heart Association/the American College of Cardiology (AHA/ACC)	2023 AHA/ACC/ACCP/ASPC/NLA/PCNA Guideline for the Management of Patients With Chronic Coronary Disease [8]	2023	Informed the content and structure of the education, and exercise training components
	Home-Based Cardiac Rehabilitation: A Scientific Statement From the American Association of Cardiovascular and Pulmonary Rehabilitation, the American Heart Association, and the American College of Cardiology [39]	2019	Identified the core components for home-based CR: patient assessment, exercise training, dietary/weight management, psychological support/management, medication adherence, and risk factor management
	Interventions to Promote Physical Activity and Dietary Lifestyle Changes for Cardiovascular Risk Factor Reduction in Adults [40]	2010	Informed the design and structure of goal setting and self-monitoring, and feedback components
British Association for Cardiovascular Prevention and Rehabilitation (BACPR)	The BACPR Standards and Core Components for Cardiovascular Disease Prevention and Rehabilitation 2023 (4th Edition) [41]	2023	Identified the core components for CR: patient assessment, health behavior change and education, lifestyle risk factor management, psychosocial health, medical risk management, and long-term strategies Informed the content of the education component
European Society of Cardiology (ESC)	2021 ESC Guidelines on cardiovascular disease prevention in clinical practice [42]	2021	Informed the exercise training component
	Standardization and quality improvement of secondary prevention through cardiovascular rehabilitation programs in Europe [43]	2020	Informed the content and structure of the education, and exercise training components
	Psychosocial aspects in cardiac rehabilitation: from theory to practice [44]	2015	Informed the content and structure of the social support component

### Conceptual model

**Figure 2:**
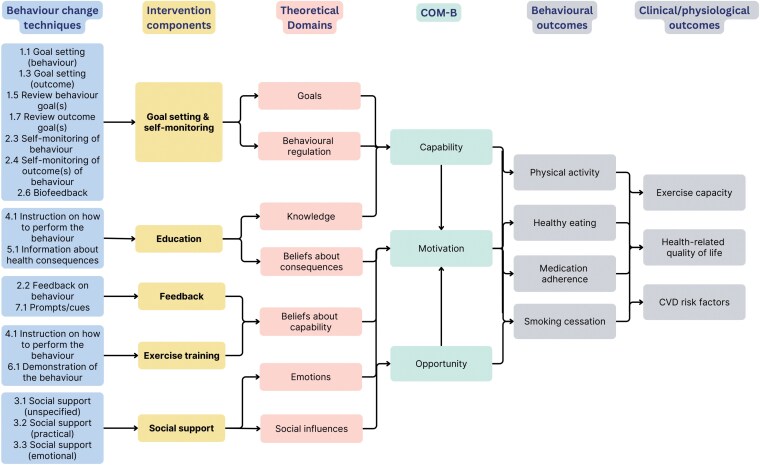
Conceptual model of digital cardiac rehabilitation

Fig. 2 depicts the conceptual model of digital CR. In summary, the model illustrates the intervention components for testing within an optimization trial, the included BCTs, the targeted theoretical domains and COM-B factors, and the behavioral and clinical outcomes of interest. The intervention components selected for inclusion encompass the core components of home-based CR as proposed by Thomas *et al*. [39]. It is important to note that while the titration and optimization of medication therapy for CVD risk factor management is an important aspect of CR, the components identified here deliberately emphasize patient self-management and lifestyle modification.

### Intervention components

#### Goal setting and self-monitoring

Health behavior change is a core component of CR as adopting healthy behaviors and maintaining lifestyle changes are essential in the prevention and control of CVD [41]. Goal setting and self-monitoring are key strategies used for behavior modification, as they can empower patients to take responsibility for their health and health-related behaviors. Evidence has shown that the use of cognitive behavioral techniques, such as goal setting and self-monitoring, in CR can lead to improvements in physical activity and diet [40, 45]. The emergence of smart wearable technologies has enabled robust monitoring of biomarkers, and behaviors important in CR (e.g. physical activity). A recent systematic review demonstrated that the use of wearable activity trackers among CR participants was associated with significant increases in daily step count and aerobic capacity [46]. In our systematic review of digital CR interventions, goal setting and self-monitoring were used in almost all the interventions (*n* = 24, 96%). The review found that BCTs related to goal setting and self-monitoring were associated with improvements in physical activity, diet, and smoking cessation. Furthermore, tele-monitoring devices, ideally suited to facilitating goal setting and self-monitoring, featured prominently in the majority of the studies (*n* = 16, 64%).

A goal-setting and self-monitoring component in digital CR should enable patients to set behavioral goals (e.g. physical activity targets) and outcome goals (e.g. target weight, blood pressure). This can be integrated into a digital platform, such as a smartphone app or web-based interface. Smart wearable tele-monitoring devices, including smartwatches and smart blood pressure monitors, can be used to enable patients to self-monitor their behaviors and vital signs. Wireless synchronization of these devices with the digital platform can facilitate the monitoring of goal progress. This component is designed to assist patients in creating clear mental representations of their desired outcomes and will provide a way of objectively managing their actions toward the attainment of their goals.

#### Feedback

Feedback from healthcare providers plays an important role in reinforcing positive behaviors and providing patients with a valuable means of gauging their progress. Existing evidence suggests that feedback is frequently included in behavior change interventions that successfully improve physical activity and diet [40]. Our systematic review reinforced the importance of feedback, with this component featuring in 22 (88%) studies. It was delivered via various channels, including email (*n* = 6, 24%), SMS messages (*n* = 5, 20%), video consultation (*n* = 5, 20%), and in-app messages (*n* = 4, 16%). In many cases, CR program staff had access to a dedicated research portal where they could view patients’ data uploads, which allowed them to personalize patient feedback.

Building upon these findings, a feedback component should consist of personalized messages to patients throughout the duration of their rehabilitation program. The messages should be designed to enhance patients’ self-efficacy, providing encouragement and guidance that drives them toward their predetermined health goals. By encouraging and empowering patients, this component is anticipated to increase patients’ motivation to perform the required health-related behaviors (e.g. physical activity, healthy eating, medication adherence, and smoking cessation).

#### Education

Patient education is a fundamental part of CR and is recommended by several international guidelines [8, 43]. According to the BACPR [41], education should be delivered to increase knowledge and also to restore confidence and foster a greater sense of perceived control. A recent systematic review underscores the positive impact of patient education on disease knowledge and the adoption of healthy behaviors among patients with coronary heart disease [47]. Our systematic review found that 17 (68%) interventions included an education component. Typically this component was presented in the form of videos and articles accessible on the study’s website or platform (*n* = 12, 48%). Alternative approaches to delivering education included SMS text messages, social media messaging (e.g. WeChat), and one-to-one tele-coaching.

Drawing from these findings, an educational component within digital CR should feature synchronous, group-based educational sessions conducted via teleconferencing. These sessions should be designed to provide patients with information on CVD, managing risk factors and leading a healthy lifestyle. The educational materials should be readily available on a digital platform for patients to review at their convenience. This component is aimed at enhancing patients’ knowledge and beliefs about the consequences of their behavior in the context of CVD. It is hypothesized that by targeting these theoretical domains, patients’ perceived capability and motivation to improve health-related behaviors (e.g. physical activity, healthy eating, medication adherence, and smoking cessation) will be increased.

#### Exercise training

Exercise training has long held a central role in CR and continues to be a cornerstone of such programs, as highlighted by clinical guidelines [39, 41, 42]. The benefits of physical activity for CVD are well established and include reductions in blood pressure, and body weight, and a positive impact on lipid profiles [48, 49]. Moreover, exercised-based CR is associated with reductions in CVD and all-cause mortality, myocardial infarction, and improvements in health-related quality of life [6].

An exercise training component in digital CR should consist of online exercise sessions delivered via teleconferencing to small groups of cardiac patients, guided by a trained exercise specialist. Following clinical guidelines, the exercise training should include both aerobic and muscular resistance exercise, individually prescribed based on pre-exercise screening and exercise testing, and entail a minimum of 36 sessions [42]. Our findings suggest that these sessions should incorporate BCTs such as instructions on how to perform the behavior and demonstrations of the behavior, while progressively increasing in difficulty. It is hypothesized that the successful completion of these exercise training sessions will enhance patients’ belief in their capability and increase motivation to continue being physically active during rehabilitation.

#### Social support

A large body of empirical evidence demonstrates that psychosocial risk factors such as social isolation, stress, depression, and anxiety increase the risk of coronary heart disease and contribute to poor health-related quality of life [44, 50]. Importantly, these risk factors can pose barriers to lifestyle changes and adherence to treatment, which can in turn moderate the effectiveness of CR [44, 51]. There is evidence that psychological interventions can reduce cardiac mortality and improve depressive symptoms, anxiety, and stress for people with coronary heart disease [52]. For these reasons, the BACPR recommends the inclusion of psychosocial health as a core component of CR [41].

Our systematic review identified 15 studies (60%) that included a component targeting psychosocial health. While these components were not always explicitly described, they often involved consultations between patients and CR program staff, focusing on reviewing progress and increasing motivation. These review sessions were done via video consultation (*n* = 3, 12%), email (*n* = 2, 8%), or phone calls (*n* = 1, 4%). Considering these findings, a component to improve psychosocial health would involve the delivery of social support through tele-consultation sessions, led by a member of the rehabilitation team and directed at small groups of patients. The focus of these sessions would be to facilitate discussions on progress, collaboratively address common problems, and provide patients with an opportunity to ask questions. This component would incorporate BCTs related to social support (e.g. unspecified, practical, emotional) and would aim to target negative emotions while boosting patients’ motivation to sustain their engagement in rehabilitation.

### Outcomes

The conceptual model proposes that the intervention components will have an impact on behavioral outcomes such as physical activity, healthy eating, medication adherence, and smoking cessation. Improvements in these outcomes will in turn influence clinical and physiological outcomes such as exercise capacity, CVD risk factors (e.g. blood pressure, cholesterol, lipid profile), and health-related quality of life.

## Discussion

CR has evolved into a comprehensive secondary prevention program that aims to stabilize or reverse the progression of CVD by managing all modifiable risk factors [53]. Yet to date, digital CR has predominantly focused on enhancing physical activity, prompting calls for more comprehensive and refined digital CR solutions [54]. This article describes the development of a conceptual model of digital CR as part of the preparation phase of MOST. The model represents the first step in delineating the intervention components to include in a comprehensive digital CR intervention and provides a framework for future correction and refinement.

The conceptual model includes candidate intervention components, BCTs, targeted theoretical domains, COM-B factors, and relevant behavioral and clinical outcomes. The candidate intervention components are designed to be comprehensive and to address the core components of CR [39, 41]. Additionally, they attempt to incorporate peer group interaction where possible, as evidence from our qualitative study and others has highlighted the importance of social interaction in digital CR [35, 55, 56]. The outcomes selected in the model include behavioral outcomes (e.g. increased physical activity, healthy eating, medication adherence, and smoking cessation), as well as clinical, physiological, and patient-related outcomes (e.g. CVD risk factors, exercise capacity, health-related quality of life). The inclusion of a number of outcomes reflects the complexity of CR as an intervention. To date, MOST studies have typically included a single primary outcome to simplify decision-making about the composition of an optimized intervention. However, recent advances in decision-making methodology in MOST have created new possibilities for interventions to be optimized based on a wide variety of decision-maker preferences [57, 58]. These developments may allow for multiple outcome variables to be considered in an optimization trial.

Following conceptual model development, the next step in this research will be to conduct pilot work to assess the feasibility and acceptability of conducting an optimization trial of the digital CR intervention. This pilot work will explore the suitability of the study design and procedures, along with the feasibility, acceptability, safety, and readiness of each intervention component. Once feasibility is established, the next step in MOST is the optimization phase, where an experimental design will be used to assess the effects of the intervention components on the targeted outcomes. By using an experimental design, it will be possible to determine the individual contribution of each intervention component. This empirical data will be used to develop an optimized digital CR intervention package that can subsequently be evaluated in an RCT.

MOST functions as a translational research framework with specific objectives for each phase of intervention development. This article serves as an example of how to meet the objectives of the preparation phase using tools and frameworks from behavioral science. The systematic review used the BCT taxonomy [32] and TIDieR checklist [34] to explore the content of digital CR interventions. This allowed the comprehensive characterization of these interventions and the identification of specific BCTs associated with behavioral improvements. The qualitative study provided the opportunity to explore the cognitions and experiences of patients who participated in digital CR. The COM-B and TDF offered a framework for understanding the potential mechanisms of change in digital CR. These tools, used in tandem, facilitated the identification of intervention components and mechanisms, demonstrating their utility in synthesizing findings to develop the conceptual model.

### Strengths and limitations

Key strengths of this development process include using multiple sources of evidence and incorporating input from key stakeholders and experts in the areas of intervention development and CR. However, several limitations must also be acknowledged. First, the coding of BCTs in the interventions was dependent on the quality of intervention reporting in published papers which was generally poor, meaning it is possible that some BCTs were not coded or were coded incorrectly. Second, the small sample size (*n* = 11) and relatively low response rate (11%) in the qualitative study, along with the sampling approach may have led to the recruitment of participants with more positive experiences. This may limit the generalizability of the findings, as a full range of participant experiences might not have been captured. A further limitation of the qualitative study is that the coding was conducted exclusively by one researcher (EK). Having a second researcher involved in this process would have strengthened the rigor of the analysis. Additionally, while the COM-B and TDF aim to encompass all the determinants of behavior change, they primarily focus on individual and psychological aspects. As a result, they do not fully account for the broader context in which participants experience the intervention. For instance, they do not capture the influence of socioeconomic, cultural, and political factors that may be unique to each participant and could have been important in the present study. Alternative methodological approaches, such as realist evaluation, could provide greater insight into how these factors influence the effectiveness of digital CR interventions. Regarding the conceptual model, the development process would have benefited from public and patient involvement; however, this was not possible due to time and resource constraints. Finally, the conceptual model focuses exclusively on patient-centered behavioral aspects of CR and does not encompass medication therapy as a component, which may be considered a potential limitation.

### Future research

The conceptual model described here marks the initial step in determining the composition of an optimized digital CR intervention and paves the way for future optimization. There is potential for future research to explore alternative delivery modalities for the intervention components. By applying MOST, it would be possible to explore the effectiveness of different modes of delivery while accounting for implementation constraints such as efficiency, affordability, and scalability. Another promising area for future research is to explore the optimal dose of digital CR. Currently, the minimum dose of CR required to reduce morbidity and mortality is unclear, with a systematic review indicating that while 36 sessions may be necessary to maximize the benefits of CR, as few as 12 sessions may still lead to improved clinical outcomes [59]. The “digital dose” of CR is even more uncertain, as it remains unclear whether substituting an in-person CR intervention with a digitally delivered version requires the same volume of sessions [54]. MOST provides a framework for answering this research question and has the potential to improve the efficiency of CR delivery.

## Conclusion

This article describes the process of developing a conceptual model of a digital CR intervention. The model identified here will serve as a blueprint for the development of a digital CR intervention and will guide decision-making in a future optimization trial. The article also serves as an example of using tools and frameworks from behavioral science to inform the preparation phase of MOST.

## Supplementary Material

ibae068_suppl_Supplementary_File

## Data Availability

Some of the materials used to conduct the systematic review are presented in a public archive: https://osf.io/bw6eq/.

## Welfare of Animals

This article does not contain any studies with animals performed by any of the authors.
