# Supplementary material for: Advancing translational research in digital cardiac rehabilitation: The preparation phase of the Multiphase Optimization Strategy
Source: Transl Behav Med. 2024 Dec 17;15(1):ibae068. doi: 10.1093/tbm/ibae068 (PMC11756285; doi:10.1093/tbm/ibae068)
Supplement: ibae068_suppl_Supplementary_File [file ibae068_suppl_supplementary_file.docx]

**Interview topic guide**

**Research study:** Exploring people’s perceptions of digital cardiac rehabilitation: a qualitative study

**Briefing:**

1. Thank participant for agreeing to take part.
2. Introduce self.
3. As described in the Participant Information Sheet we are interested in finding out people’s experience of participating in a digital cardiac rehabilitation programme.
4. If at any time during the interview you do not wish to answer a question that’s okay.
5. I would like to record our conversation. The recording will be typed out, but everything you say will be anonymous. Your name and any names or places you mention will be taken out, so that if someone read your interview they would not know who you are.
6. If, at any stage, you wish to stop the audio recorder, please let me know.
7. Do you have any questions?

**Topics to be explored:** Below is a list of questions to be discussed in this study. The work will remain flexible with respect to participants’ agendas but we will cover the main topics outlined below. It is common in semi-structured work to develop topics and questions as new ideas emerge from early data collection. Therefore, we may add new topics as the interviews progress and data collection continues. However, the key topic of exploring people’s experience of participating in a digital cardiac rehabilitation programme will remain the focus of the interview.

**1. Patient Journey**

1. When was your first cardiac event?
2. Could you tell me about your cardiac history in recent times?

**2. Views on the programme**

1. What was your overall experience of the Croí MySláinte/Our Hearts Our Minds programme?
2. Did the programme lead to any *changes* in your life? Could you give examples?

**Prompt:** physical health; mental health, risk factors, quality of life

*(make a note of the changes mentioned - follow up each with the next two questions)*

- 1. What parts of the programme helped you to change X?
  2. Why do you think the programme helped with that?

**3. Views on the mode of delivery**

1. What did you think about how the online delivery of the programme?

**Prompt:** Ease of use; engagement; ability to get support

**4. Feedback on the programme**

1. What could have been better about the Croí MySláinte/Our Hearts Our Minds programme?

**Prompt:** Anything that did not work well? Anything that could have been added to or removed from the programme? Anything that needed more time/emphasis?

**4. Summary**

1. Was there anything I left out?
2. Anything else you would like to tell me?
